# Evaluation of the pneumococcal urinary antigen test (PUT):
a retrospective study

**DOI:** 10.20407/fmj.2019-028

**Published:** 2020-07-14

**Authors:** Tatsuyoshi Yokoi, Kazunobu Kuwabara, Kiyotaka Ono, Yusuke Kito, Kenichi Kato, Keisuke Kato, Masahiro Hirose, Rieko Kondo, Takahiko Horiguchi

**Affiliations:** Department of Respiratory Medicine II, Fujita Health University, School of Medicine, Nagoya, Aichi Japan

**Keywords:** Pneumococcal pneumonia, Urinary antigen testing, *Streptococcus pneumoniae*, Bacterial pneumonia

## Abstract

**Objectives:**

To determine the usefulness of the pneumococcal urinary antigen test (PUT) and to describe the characteristics of pneumococcal pneumonia.

**Methods:**

In this retrospective study, we examined the effects of prior antibiotic treatment, pneumonia onset period, and sputum quality on the results of PUT. Clinical information was collected via medical records from all adult patients who were hospitalized at the Fujita Health University Bantane Hospital with “pneumonia” as a new diagnosis from April 2015 to March 2018.

**Results:**

A total of 482 patients with pneumonia were included, of whom 103 had pneumococcal pneumonia. The frequency of PUT positivity did not differ significantly in patients with a pneumonia onset period of ≥3 days compared with those with a period of ≤2 days (*P*=0.514). Patients with a history of prior antimicrobial therapy had a significantly lower rate of positive sputum culture vs those with no such history (*P*=0.005); however, PUT positivity in the two groups did not differ significantly (*P*=0.367).

**Conclusions:**

Our results showed that urinary antigen testing for pneumococcal pneumonia is useful for diagnosis regardless of prior antibiotic treatment and time since symptom onset.

## Introduction

Bacterial pneumonia is a common, but serious, respiratory infection with a high prevalence. This is especially true in Japan, where a large proportion of the population is elderly and where pneumonia is the third leading cause of death.^1^ Given the high prevalence, pneumonia management is an important issue. Pneumonia has many microbial causes, including *Streptococcus pneumoniae*, *Haemophilus influenzae*, and *Mycoplasma*, and *Legionella*. *S. pneumoniae* is the most common cause of both community-acquired pneumonia (CAP) and nursing and healthcare-associated pneumonia (NHCAP). Pneumococcal pneumonia, caused by *S. pneumoniae* tends to be severe and has a high mortality rate in older populations.^2^

The treatment for bacterial pneumonia is antibacterial therapy, and the drug selection varies depending on the bacterial species and the organism’s drug sensitivity. Tests performed to identify the causative organism include sputum and blood culture, urinalysis, and immunological assay. A drawback of sputum culture is that it requires a high-quality sample to ensure accurate identification of a causative organism in the lower respiratory tract. Microscopic evaluation of sputum is performed according to the Geckler classification, and specimens containing too many epithelial cells and/or too few white blood cells are unsuitable for viewing under low magnification. Generally, Geckler classification grade 4 or higher (>25 leukocytes and <10 squamous cells in a Gram-stained ×100 field of view) is considered a good quality smear, and the culture results of sputum samples that do not meet these standard results are considered unreliable.^3^ Despite the importance of a high-quality culture in the accurate identification of causative bacteria in pneumonia cases, only 14%–28% of acute pneumonia cases can be identified by a high-quality sample.^4,5^ This may be a result of both the difficulty of collecting a lower respiratory specimen as well as the fact that patient may have received prior antimicrobial therapy. In such cases, correct bacterial culture results cannot be obtained because of changes in the bacterial flora.^6–8^

The pneumococcal urinary antigen test (PUT) is a rapid diagnostic method to identify the causative microorganism after pneumonia has been diagnosed. PUT is a rapid detection kit for pneumococcal pneumonia that was developed to detect the causative bacteria in urine specimens and was approved by the US Food and Drug Administration in 1999. BinaxNOW *Streptococcus pneumoniae*^®^ (Binax Inc., Waltham, MA) is a PUT that detects capsular polysaccharide antigen, a cell wall component of *S. pneumoniae* excreted in urine, using an immunochromatographic membrane assay. PUT provides results within 15 minutes and is reported to have a sensitivity of 50%–80% and a specificity of >90% for diagnosing pneumococcal pneumonia.^9–11^ This test is widely used in the management of pneumonia.^12^ In Japan, the cost of PUT has been covered by the National Health Insurance network since 2005. The British Thoracic Society’s community pneumonia guidelines state that all patients with moderate or severe CAP should undergo PUT,^13^ and Japanese guidelines for pneumonia also recommend urinary antigen testing for patients with CAP.^14^

The PUT result can be delivered regardless of the quality of the specimen. According to the test method, prior antibiotic therapy is considered to have little effect on sensitivity. However, some reports state that prior antibiotic therapy reduces the sensitivity of PUT,^15,16^ and a 2013 systematic review of urinary pneumococcal tests concluded that the effect of prior antibiotic treatment on the sensitivity of PUT is unclear.^17^

The BinaxNOW *Streptococcus pneumoniae*^®^ package insert states that it takes 3 days from the onset of symptoms until the amount of urinary pneumococcal capsular antigen exceeds the detection sensitivity of the PUT. Therefore, it is possible to have a negative urinary antigen test result immediately after the onset of pneumonia symptoms. Although this problem is clinically important, few reports have evaluated the period from the onset of pneumonia to PUT.

The purpose of this study was to evaluate the usefulness of PUT for CAP and NHCAP, and to examine how the results are affected by prior antibiotic treatment, symptom duration, and sputum quality. We addressed this objective in this retrospective study of patients with bacterial pneumonia admitted to our hospital.

## Methods

### Study population

We analyzed data from all adult patients who were hospitalized from April 2015 to March 2018 at the Fujita Health University Bantane Hospital (a 370-bed university teaching hospital in Nagoya, Aichi, Japan) with “pneumonia” as a new diagnosis. Clinical information was collected retrospectively via patients’ medical records.

**Figure 1:**
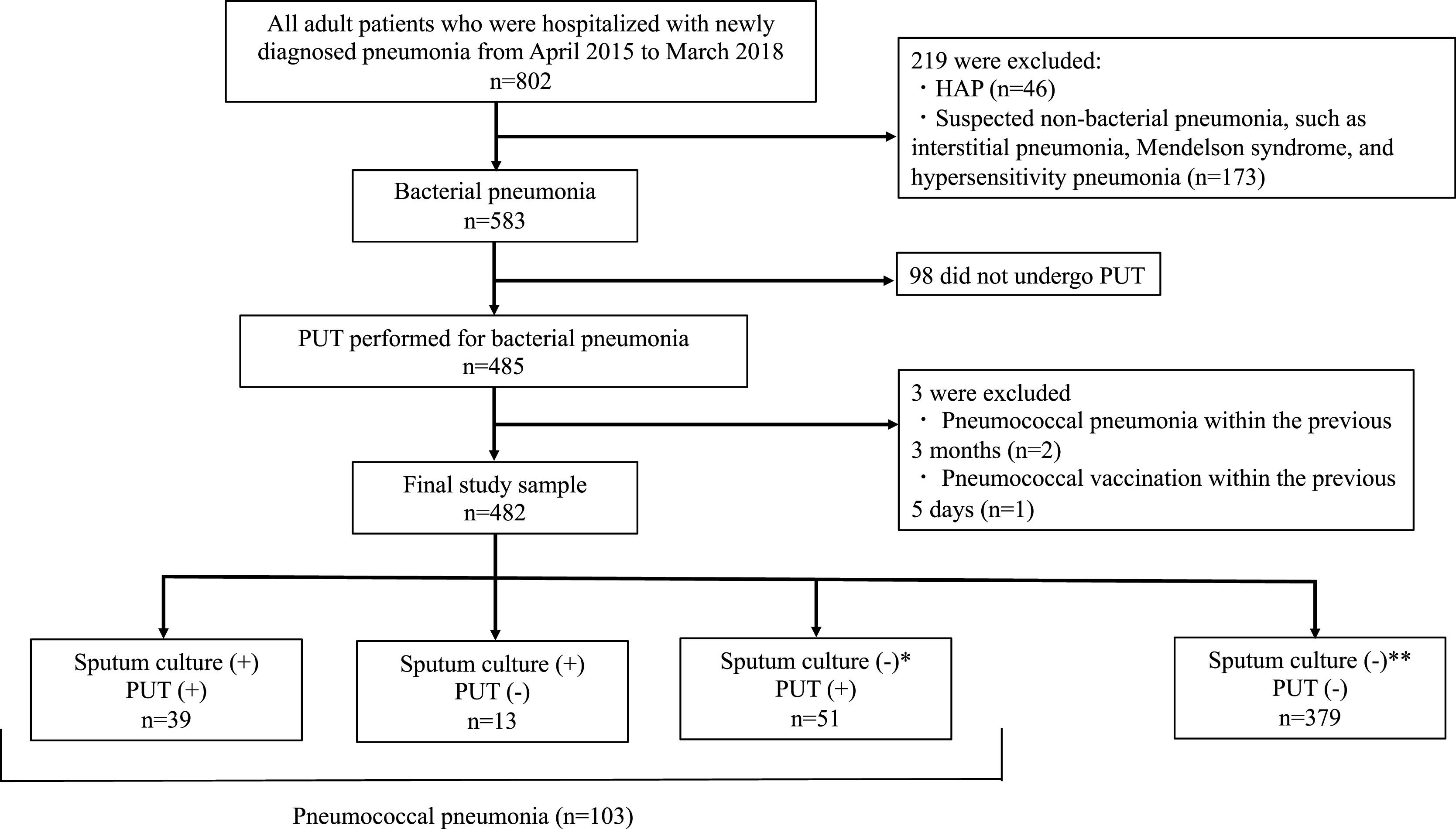
Flowchart showing the participant selection process HAP, hospital-acquired pneumonia; PUT, pneumococcal urinary antigen test ^†^ Including one patient without sputum culture test results. ^‡^ Including eight patients without sputum culture testing.

A flowchart of patient selection in this study is shown in Figure 1. We made the following exclusions: patients with hospital-acquired pneumonia and non-bacterial pneumonia; patients for whom PUT and sputum culture were not performed on the day of admission; patients with a history of pneumococcal pneumonia within the previous 3 months; and those who had received pneumococcal vaccination within the previous 5 days.

### Definitions

CAP was defined as radiographic evidence of an infiltrate and at least one of the following symptoms: fever, cough, sputum production, dyspnea, and pleuritic pain.

Patients with interstitial pneumonia or pulmonary tuberculosis were excluded from the study. Pneumococcal pneumonia was defined as either positive sputum culture for *S. pneumoniae*, or a positive PUT result. NHCAP, hospital-acquired pneumonia, and CAP were defined according to Japanese Respiratory Society guidelines.^14^ We defined onset time as the day acute pneumonia-related symptoms developed. Symptoms of acute pneumonia were defined by respiratory symptoms as well as by systemic manifestations such as malaise and anorexia. For those with multiple onset days in the medical record, we used the day most distant from the time of admission as the onset date, in our analysis. Data regarding previous antibacterial drug administration were collected from patients’ medical records and from information provided by other hospitals.

Prior antibacterial drug use was confirmed through patients’ medical records, and we excluded patients for whom it was not possible to determine whether the type of prior drug was an antibacterial drug.

The quality of sputum used for the sputum examination was determined according to the Geckler classification, and samples with >25 leukocytes and <10 squamous epithelial cells (Geckler grade 4+) in a Gram-stained ×100 field were considered good quality.

This study was approved by the Medical Research Ethics Committee of Fujita Health University (Approval No. HM18-146). The study conformed to the principles outlined in the declaration of Helsinki, and the need for informed consent was waived because the study was retrospective.

### Statistical analysis

We compared two independent groups using the Mann–Whitney U test. Data were compared between groups using Pearson’s Chi-square test and analysis of discrete variance, as appropriate. Continuous data were expressed as means and standard deviations, or medians and interquartile ranges, and categorical data were expressed as counts and percentages. *P*-values <0.05 were considered statistically significant. StatMate version 3.19 (ATMS Co., Ltd., Tokyo, Japan) was used for all statistical analyses.

## Results

### Patients

During the study period, 802 patients were hospitalized with a diagnosis of “pneumonia”. We excluded patients with non-bacterial conditions (n=219) such as interstitial pneumonia and hypersensitivity pneumonia, and included 583 patients with acute pneumonia. Among the 583 patients with acute pneumonia, 83.2% (n=485/583) underwent PUT. After excluding patients who did not receive PUT (n=98), those reporting pneumococcal vaccination within 5 days (n=1), and those with a history of pneumococcal pneumonia within 3 months (n=2), we included data for the remaining 482 patients in the analysis. Patients in the final cohort had one of two types of pneumonia: pneumococcal or non-pneumococcal. Pneumococcal pneumonia accounted for 21.4% of patients (n=103/482). Patients’ backgrounds are shown in Table 1.

### Microbiological test results

A total of 103 patients had pneumococcal pneumonia, of whom 37.9% were sputum culture (+), PUT (+); 12.6% were sputum culture (+), PUT (−); and 49.5% were sputum culture (−), PUT (+) (Fig. 1). Of the patients diagnosed with pneumococcal pneumonia, 90 (87.4%) were urinary pneumococcal antigen-positive, and 52 (50.5%) were sputum culture-positive. Of the 52 strains obtained from sputum culture, 3 were unable to undergo drug sensitivity testing because of poor growth. All other strains had a penicillin G-minimum inhibitory concentration ≤2 μg/mL, and were identified as penicillin-susceptible *S. pneumoniae*.^18^

### Pneumonia onset and test results

We examined the relationship between pneumonia onset time and test results for 97 patients whose period from onset to microbiology could be determined (Table 2). Patients were divided into two groups according to their time from pneumonia onset to microbial testing as <3 days (n=36) or ≥3 days (n=61). The rate of sputum culture positivity did not differ significantly between patients with an onset period of ≥3 days compared with those with an onset period of <3 days (38.9% vs 59.0%, respectively; *P*=0.058). Similarly, the rate of urinary antigen positivity did not differ significantly between the two groups (88.9% vs 85.2%, ≥3 days vs <3 days, respectively; *P*=0.514).

### Prior antibiotic treatment

A history of prior antibiotic treatment was noted in 28.8% (n=137/475) of the patients, and there was no significant difference between the two groups regarding prior antibiotic use (25.2% vs 29.8%, ≥3 days vs <3 days, respectively; *P*=0.420) (Table 1). We divided patients with pneumococcal pneumonia into groups with and without prior antimicrobial therapy (Table 2). Compared with those without prior antimicrobial therapy, patients with prior antimicrobial therapy had a significantly lower frequency of pneumococcus positivity in sputum culture (30.8% vs 57.1%, respectively; *P*<0.005), but there was no significant difference in urinary pneumococcus positivity status (92.3% vs 85.7%, respectively; *P*<0.367).

### Sputum quality

Of the 482 patients, 473 (98.1%) underwent a sputum culture test on admission. Excluding two specimens that could not be evaluated by the Geckler classification, 29.9% (n=141/471) of the specimens were of good quality (Geckler grade 4+) (Table 1). Patients in the pneumococcal pneumonia group produced good quality sputum more frequently than those in the non-pneumococcal pneumonia group. The frequency of producing good sputum quality did not differ significantly between patients in the pneumococcal pneumonia group and those in the non-pneumococcal pneumonia group (37.3% vs. 27.9%, respectively; *P*=0.054).

We classified sputum used for culture into two groups: Geckler 1–3 and Geckler 4–6, according to the number of leukocytes and squamous cells contained in the culture (Table 2). Pneumococcal culture positivity was significantly higher in the Geckler 4–6 group than in the Geckler 1–3 group (60.5% vs 39.1%, respectively; *P*=0.046). Urinary pneumococcal antigen positivity did not differ significantly between the two groups (87.5% vs 86.8%, Geckler 1–3 vs Geckler 4–6, respectively; *P*=0.995).

## Discussion

Pneumococcal pneumonia accounted for 21.4% of the hospitalized pneumonia patients in our sample. These results are similar to the frequency of pneumococcal pneumonia found among Japanese patients with acute pneumonia (18.8% for CAP and 17.3% for NHCAP).^14^ In the present study, PUT was performed in 83.2% of the patients hospitalized for acute pneumonia. This test is used often in clinical practice because of its rapidity and ease of sample collection.

In this study, we found no difference in urinary pneumococcal antigen positivity between early (<2 days) and late (>3 days) symptom onset. The BinaxNOW *Streptococcus pneumoniae*^®^ package insert states that urinary capsular discharge can be detected as early as 3 days after symptom onset. However, in our study, PUT detected pneumococci within 3 days of onset, indicating that urinary capsular antigen discharge begins within 2 days of symptom onset. Similar to our results, Fukushima et al. found urinary pneumococcal antigen positivity even in patients for whom the period between pneumonia onset and the urinary antigen test was <2 days.^19^ These results indicate that, in patients with severe pneumonia who are hospitalized, urinary excretion of capsular antigens begins early after the onset of symptoms, and that the presence of early-onset acute pneumonia is not necessarily a reason to avoid using urinary antigen tests to detect pneumococcal pneumonia.

The frequency of pneumococci isolation by sputum culture was lower in patients with a history of prior antibacterial therapy than in those without. Additionally, studies have confirmed that the bacterial flora in sputum can change secondary to prior antibacterial therapy, and that pneumococci in sputum can be difficult to culture in patients with prior antibacterial therapy.^5,7,8^

PUT is considered less susceptible to antibacterial therapy because it detects the capsular polysaccharide antigen by immunochromatographic membrane assay. However, some reports state that prior antibiotic therapy reduces the sensitivity of PUT;^15,16^ therefore, the effect of prior antibiotic treatment on the sensitivity of PUT is unclear.^17^

In our study, we found no significant difference in PUT positivity for patients with a history of prior antimicrobial therapy compared with those without (92.3% vs. 85.7%, respectively; *P*=0.367). This may be because PUT detects the capsular polysaccharide antigen, a cell wall component of *S. pneumoniae* excreted in urine, using an immunochromatographic membrane assay.

In the present study, we divided patients with pneumococcal pneumonia according to their Geckler sputum specimen classifications. The rate of pneumococcal positivity in sputum culture was significantly higher in the Geckler 4–6 group than in the Geckler 1–3 group, but the urinary pneumococcal-positive rate did not differ significantly between the two groups. Geckler et al.^3^ reported that quality control of sputum using Gram staining was performed with the appropriate quantity of polynuclear leukocytes and squamous epithelium, and that *S. pneumoniae* could be detected in good-quality sputum samples in adult patients with CAP. Studies report that a good-quality sputum sample can be obtained in 14%–28% of patients with acute pneumonia,^3,4^ and in our study, a good-quality sputum sample was obtained in 29.9% of all patients with acute pneumonia. The results of our study are almost identical to previous reports, indicating that a good-quality sputum sample can be obtained in only a small proportion of patients with pneumonia. Because many patients with acute pneumonia have low-quality sputum samples at the time of hospitalization, reliable sputum cultures are often not obtained. PUT may be a useful alternative for diagnosing pneumococcal pneumonia in these patients.

False-positive results are possible with PUT in the following circumstances: pneumococcal infection within 3 months of testing, pneumococcal vaccination, pneumococcal carriage in children’s nose and pharynx,^20^ and in *S. mitis* cross-antigenicity. We identified no patients in our study with *S. mitis* detected in sputum culture, and a previous report shows that this organism does not affect PUT results, clinically.^21^ The frequency of pneumococcal colonization decreases with age and is reported to be <4% in adults.^22^ Therefore, PUT positivity secondary to colonization is a problem for children, but is not a problem in adults. Additionally, Marcos et al.^23^ reported no PUT positivity in adults with HIV infection despite colonization of *S. pneumoniae* in the nasopharynx, secondary to immunosuppression. All patients in our study were adults, and it is probable that few carried pneumococci.

### Limitations

Our study had limitations. First, because of the retrospective design, data for some patients were not available. Second, although patients with urinary pneumococci-positive results were not included in this study, the urinary antigen test was performed at the discretion of the doctor in charge; therefore, patients’ backgrounds may be biased. Finally, the causes of false-positive urinary pneumococci results include pneumococcal pneumonia within the previous 3 months and pneumococcal vaccination within the previous 5 days. In this study, we excluded patients meeting either of these conditions; however, some patients meeting these conditions may not have been excluded because this was a retrospective study, and these patients’ medical records may have been incomplete.

## Conclusion

Our results showed that the urinary antigen test for pneumococcal pneumonia can produce accurate results regardless of prior antibiotic treatment, time since symptom onset, and the quality of the sputum sample. This is in contrast to the sputum test, which was affected by both prior antibiotic treatment and the quality of the sputum sample. Accordingly, we suggest that urinary antigen testing for pneumococcal pneumonia is more useful for diagnosis. This study included only hospitalized patients with pneumonia; therefore, a study including patients with mild pneumonia managed as outpatients is required.

## Figures and Tables

**Table1:** Patients’ characteristics according to pneumococcal pneumonia status

	All (n=482)	Pneumococcal pneumonia (n=103)	Non-pneumococcal pneumonia (n=379)	P-value
Male gender, n (%)	257 (53.3)	54 (52.4)	203 (53.6)	0.838
Age (years), mean±SD	74.3±18.0	76.6±16.6	73.6±18.4	0.121
Age (years), n (%)				
<45	48 (10.0)	7 (6.8)	41 (10.8)	0.227
45–64	46 (9.5)	10 (9.7)	36 (9.5)	0.949
65–84	236 (49.0)	50 (48.5)	186 (49.1)	0.924
≥85	152 (31.5)	36 (35.0)	116 (30.6)	0.400
Infection source^†^				
CAP:NHCAP, n(%)	309 (67.0):152 (33.0)	56 (56.6):43(43.4)	253 (69.9):109 (30.1)	0.012*
High-quality sputum collection^‡^	141 (29.9)	38 (37.3)	103 (27.9)	0.054
Prior antibiotic administration^§^	137 (28.8)	26 (25.2)	111 (29.8)	0.420

^†^ We excluded patients whose source of infection could not be determined (n=21). ^‡^ Excluded: Sputum culture was not performed (n=7); the date of examination was not the date of hospitalization (n=1); the sputum quality was indeterminate (n=3). ^§^ Excluded: Treated with an unknown drug (n=7). CAP, community-acquired pneumonia; NHCAP, nursing and healthcare-associated pneumonia; SD, standard deviation

**Table2:** Factors associated with positive *Streptococcus pneumoniae* culture and positive pneumococcal antigen test results among patients with pneumococcal pneumonia (n=103)

Factor	Sputum culture positive				PUT positive			
	Proportion (%)	P-value	Odds ratio (95%CI)	Adjusted P-value	Proportion (%)	P-value	Odds ratio (95%CI)	Adjusted P-value
Delay between pneumonia onset and performing the test^†^		0.055	0.41 (0.16–1.03)	0.058		0.760	1.54 (0.42–5.69)	0.514
<3 days	14/36 (38.9)				32/36 (88.9)			
≥3 days	36/61 (59.0)				52/61 (85.2)			
Prior antimicrobial therapy		0.020*	0.20 (0.06–0.60)	0.005**		0.381	2.13 (0.41–10.90)	0.367
Prior antimicrobial therapy	8/26 (30.8)				24/26 (92.3)			
No prior antimicrobial therapy	44/77 (57.1)				66/77 (85.7)			
Quality of the sputum sample^‡^		0.036*	2.78 (1.02–7.55)	0.046*		0.923	1.00 (0.28–3.57)	0.995
Geckler 4–6	23/38 (60.5)				33/38 (86.8)			
Geckler 1–3	25/64 (39.1)				56/64 (87.5)			

PUT, pneumococcal urinary antigen test ^†^ We excluded six patients because the time of symptom onset was unknown. ^‡^ We excluded one patient because the sputum culture results could not be evaluated.
